# Causes of delayed outbreak responses and their impacts on epidemic spread

**DOI:** 10.1098/rsif.2020.0933

**Published:** 2021-03-03

**Authors:** Yun Tao, William J. M. Probert, Katriona Shea, Michael C. Runge, Kevin Lafferty, Michael Tildesley, Matthew Ferrari

**Affiliations:** ^1^ Intelligence Community Postdoctoral Research Fellowship Program, Oak Ridge, TN, USA; ^2^ Department of Ecology, Evolution and Marine Biology, University of California, Santa Barbara, CA, USA; ^3^ US Geological Survey, Western Ecological Research Center at Marine Science Institute, University of California, Santa Barbara, CA, USA; ^4^ Big Data Institute, Li Ka Shing Centre for Health Information and Discovery, University of Oxford, Oxford, UK; ^5^ Department of Biology, 208 Mueller Laboratory, Pennsylvania State University, University Park, PA, USA; ^6^ The Center for Infectious Disease Dynamics, Pennsylvania State University, University Park, PA, USA; ^7^ US Geological Survey, Patuxent Wildlife Research Center, Laurel, MD, USA; ^8^ The Zeeman Institute for Systems Biology and Infectious Disease Epidemiology Research, School of Life Sciences and Mathematics Institute, University of Warwick, Coventry, West Midlands, UK

**Keywords:** foot-and-mouth, epidemics, outbreak management, livestock diseases, response delay

## Abstract

Livestock diseases have devastating consequences economically, socially and politically across the globe. In certain systems, pathogens remain viable after host death, which enables residual transmissions from infected carcasses. Rapid culling and carcass disposal are well-established strategies for stamping out an outbreak and limiting its impact; however, wait-times for these procedures, i.e. response delays, are typically farm-specific and time-varying due to logistical constraints. Failing to incorporate variable response delays in epidemiological models may understate outbreak projections and mislead management decisions. We revisited the 2001 foot-and-mouth epidemic in the United Kingdom and sought to understand how misrepresented response delays can influence model predictions. Survival analysis identified farm size and control demand as key factors that impeded timely culling and disposal activities on individual farms. Using these factors in the context of an existing policy to predict local variation in response times significantly affected predictions at the national scale. Models that assumed fixed, timely responses grossly underestimated epidemic severity and its long-term consequences. As a result, this study demonstrates how general inclusion of response dynamics and recognition of partial controllability of interventions can help inform management priorities during epidemics of livestock diseases.

## 1. Introduction

Despite growing international participation in a concerted effort to prevent notifiable livestock diseases [1,2], mass outbreaks continue to occur globally, causing significant losses to both national economies and human lives as well as concerns over animal welfare. In recent examples, the 2000 Saudi Arabia outbreak of Rift Valley fever (RVF) led to a more than 40% reduction in trade value in sub-Saharan African regions [3]. Recurrences of foot-and-mouth disease (FMD) outbreaks in FMD-free countries incur an annual cost of US$1.5 billion worldwide and an order of magnitude more in endemic countries [4,5]. Pandemic influenza A H1N1, aided by intercontinental pig trade, was responsible for an enormous human death toll in Asia and Africa [6]. Many animal health services and protocols designed to control livestock disease spread have been shown to underperform relative to management expectations [7,8]. Meanwhile, protracted implementations of outbreak response policies (e.g. livestock culling, heavy trade restrictions) have led to strong public opposition and further destabilization of global market signals in some cases [9,10]. The emergence and spread of livestock diseases are expected to accelerate due to climate change and increasingly intensified agricultural practice (e.g. [11]). Thus, understanding why control actions often fail to deliver the desired outcomes remains a critical challenge in the effort to improve future responses.

Depopulation of infected premises has been a well-established management practice for stamping out notifiable livestock diseases [12–14]. Imposing a short turnaround time from notifying infection on properties to depopulating infected areas aids in preventing an outbreak from getting out of control [15,16]. Temporary viability of pathogens after host death raises further concern about their residual transmission potential through carcasses and fomites [17,18]. In response, control guidelines have been strengthened to include additional, post-culling processes of disposal (e.g. carcass burning and rendering) and decontamination. Short completion times of these downstream control actions would also contribute to the provision of a strong biosecurity programme [19].

Depopulation and disposal efforts are often carried out slowly across individual farms (see [20]). Response delays (e.g. time from reporting infected holdings to slaughtering the animals) can be partially attributed to logistical constraints that exist across management infrastructures. They include added wait-time for veterinary virologists to confirm infection diagnoses [21], delay to legally procure labour, supplies, transportation and control facilities [22], poor accessibility to infected properties that are geographically remote [23], a shortage of personnel and resources [24], non-compliance with control measures stemming from monetary disputes between the government and land occupiers seeking compensation [9] and the unsustainability of existing control methods after negative public reactions [19,25]. Logistical problems also arise from positive feedback between transmission and management load. An increased strain on operational capacity owing to the rapid spread of infection can create a growing backlog of pending cases, with newly reported premises left untreated for extended periods [26]. Due to this complex ‘human element’ [27] in carrying out responses to local outbreaks, delays in interventions on targeted premises are difficult to predict and control amid an epidemic.

Mathematical models have been critical to the development of realistic outbreak predictions and effective intervention strategies. Local responses are commonly modelled to follow a predetermined schedule that is uniform across farms within set dates (e.g. [13,28–30]). Recent simulation models (e.g. [31,32]) incorporated more complex response processes by accounting for hypothetical effects of resource capacity on the local efficiencies of control operation. However, without close examination of actual management data, the constraining factors behind individual instances of response delay remain a major source of uncertainty that, if misrepresented, may mislead epidemic projections and the choice of optimal control strategies.

We revisit the 2001 FMD epidemic in the UK as a case study for response time variation during a livestock disease outbreak. We focus our analysis on this outbreak and its response campaign for three principal reasons. First, it was extensively documented, thus providing fine-scale schedule and location data on individual control actions taken. Second, the epidemiological process (e.g. high contagiousness, density-dependent transmission rate, farm-level control, potential viability of infectious agents in carcasses) shares key features with sheep scrapie, avian influenza, hog cholera, RVF and more, allowing the results to be informative for livestock diseases in general. Third, the 2001 case has motivated the development of numerous epidemiological models [33], which we use as the basis for our present analysis.

We examined the management timeline of this historic epidemic and estimated the effects of general logistical factors on culling and disposal delays to individual farms. We subsequently simulated delay times within different operational contexts and evaluated how increasingly accurate model representations of the response process influenced outbreak predictions. By addressing the causes and consequences of response delays, we show how commonly neglected features of livestock disease management may affect management expectations and how incorporating these features in models can better predict future outbreaks.

## 2. Methods

We used individual farm records collected by the Department for Environment, Food and Rural Affairs during the 2001 FMD outbreak in the UK. The line list contains premises identifiers in the form of county-parish-holding (CPH) number, the coordinates of the farmhouse, UK grid reference, date of infection report, end date and hour of slaughter, end date and hour of carcass disposal and the number of livestock (cattle, pigs, sheep and goats). We focused our analysis of response delays on only the premises initially identified as infected (IP) and culled to limit viral excretion. This excludes ‘at-risk’ premises that were culled pre-emptively for being in direct, dangerous contacts with IPs (DC), contiguous to IPs (contiguous premises, CP), in neighbourhood of IPs (3–5 km rings) or suspicious (slaughter-on-suspicion, SOS), but did not test positive for the virus. These premises were excluded because the initial date of the decision to cull was not recorded. We also included in our analysis farms that were initially considered ‘at-risk’ but were later reclassified as IP (e.g. 162/8196 of DC, 71/335 of SOS) with known infection report dates. 171 out of 2021 entries whose date fields were incomplete or inconsistent with the sequence of report–slaughter–disposal were discarded from analysis. For premises composed of multiple parcels or fragments of land that share the same CPH numbers, we aggregated livestock quantity and response times for large parcels (containing 50 animals or more) and discounted entries for small, remnant parcels. From this processed dataset, we calculated the time intervals between report-to-culling completion (defined as culling delay) and culling completion to disposal completion (defined as disposal delay) in fractional days for each infected farm, e.g. perfect compliance with the national policy to depopulate IPs under 24 h of report would nevertheless return a positive culling delay that equals the number of operational hours in unit days. Activities that were recorded without hourly information were set to occur at midnight at the start of the recorded day.

To identify key logistical constraints, we applied survival analysis to the culling and disposal delays using Cox proportional hazards regression, implemented using the R package coxme [34]. We explored the logistical effects of three candidate covariates: farm size, control demand and farm density. Farm size counts all livestock on the farm to be culled, in units of one hundred. It is possible that highly populated premises experience longer response times due to a positive relationship between the number of animals handled and labour hours [35]. Control demand measures the national number of premises that are scheduled for control, in units of ten. This relates to the backlog of farms awaiting control at a particular time, which is directly limited by operational capacity [22,36]. In our analysis of culling delay, the demand covariate tallies pending cases (i.e. infected premises that are not yet culled) on the day of the focal farm's case report. Alternatively, in the analysis of disposal delay, the demand covariate tallies premises pending disposal (i.e. culled but with carcasses remaining) on the day of the focal farm's culling completion. We note that these measures use IP caseload as a proxy of the overall demand on the response system, excluding pre-emptive culls and disposals that lack explicit documentation of time in-and-out of the control queue. Farm density is defined as the number of IPs in units of ten within a geographical neighbourhood of 5 km radius, which we computed using the R package spatstat [37]. Clustering of farms is correlated with infection risk [13], but it may also influence the accessibilities of remote, isolated premises and their management priorities, leading to variable response times. County membership was assigned as a random effect, which allows us to account for regional variation in operational conditions and management practices. In addition, we ran separate regression analyses on farms that were reported before (*N* = 830) and on or after (*N* = 970) 1 April, close to when the national control policy was strengthened by the Ministry of Agriculture, Fisheries and Food (MAFF) with a target schedule of culling IPs and associated ‘at-risk’ farms within 24 and 48 h of case reporting, respectively.

In order to assess the epidemiological impacts of realistic response times, we simulated FMD outbreaks subject to context-dependent control actions. The durations of delay were generated according to the method of inverse probability integral transform [38]. The baseline hazard assumed the commonly used Weibull distribution, which offers flexibility in modelling a variety of survival data [39]; its shape and scale parameters *k* and *λ*, respectively, were estimated via model fitting using the R package flexsurv [40]. Delay time *T* is a random variable distributed as a conditional survival function derived from the proportional hazards regression model. Each realization was obtained by computing

2.1 $$t\;=\;{\left(-\frac{\log(v)}{\lambda \exp(x^{\prime }\beta )}\right)}^{1/k},$$

with *v* a uniform variate on (0,1), **x** is a vector of covariates (i.e. farm size, control demand, farm density) and *β* is the logarithm of their corresponding hazard ratios (HRs), which measures the relative change in hazard rate as the value of a covariate increases. The censoring time was set uniformly at the maximal recorded value (42 days). We then tested our delay predictions by applying parametric bootstrap based on estimations of mean HRs of key covariates from 2000 simulations replicates, each generated 1000 delays using covariate values randomly sampled from the national data. The mean HRs from our predictions were then evaluated against the empirical estimates.

We integrated the predicted response delays into the well-established Warwick model [27,41–44]. The model includes spatially explicit representation of registered farms and their livestock compositions. It treats the farm as the basic unit for infection and susceptibility, such that all the animals in each holding become infected *en masse*. The parameters are fitted to the incidence data from 2001 and account for nonlinear increases of farm-level transmission and susceptibility as a function of farm size. Here, we extended the model description of control actions to include the disposal process, carcass transmission rate, and variable culling and disposal delays that equate to an individual farm's wait-times in the control queues. The viral excretion ratio between living and dead animals in FMD has been seldom investigated; for the purpose of this analysis, we assumed that the carcass transmission rate was 10% of the baseline rate established prior to culling. As we are principally interested in the predictive differences resulting from the introduction of response delays rather than a recreation of the 2001 outbreak, the original model was not reparametrized under these new features.

We considered a general scenario where only IPs and DCs are targeted for removal. Two operational settings were explored: the time-independent response predicts delays using a set of HRs estimated over the entire epidemic timeline; the time-dependent response accounts for the effect of the policy change and uses separate sets of HR estimates, one limited to farms reported before 1 April and the other to those reported afterwards. We compared their epidemic outcomes to simulation runs under two alternative delay scenarios: (i) idealized response characterized by constant, uniform delays, such that culling is completed under 24 h for IP and 48 h for DC, and disposal is completed under 24 h for IP and 24 h for DC, to reflect the target response time for culling adopted by MAFF and a conservative ideal for disposal response times given that there was no national policy for disposal delay; (ii) approximated response, in which case delays are drawn randomly from IP empirical distributions and an extra 24 h is added to DC culls to adjust for their later occurrences. The latter scenario reflects the true distribution of delays, but not the observed correlations between covariates and delay times.

## 3. Results

**Figure 1.**
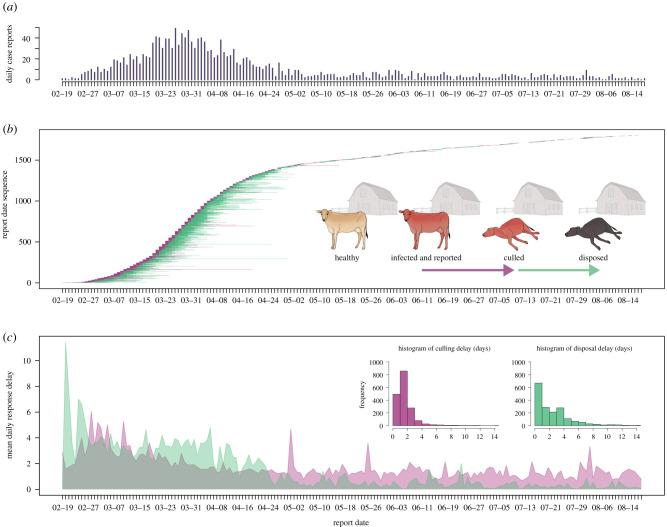
Temporal variations in response delays on IPs during the 2001 foot-and-mouth epidemic in the UK. (*a*) Incidence time-series based on daily national case reports. (*b*) Delay intervals from infection report to culling (purple) and from culling to disposal (green) for each farm with respect to its report date. Illustration credit: Life Science Studios. (*c*) Mean delays to culling (purple) and disposal (green) as a function of report date; the insets show the overall distributions of both delays.

The culling and disposal delays of IPs in the 2001 FMD outbreak were highly variable, and frequently longer than the operational recommendations of 24 h issued on 1 April (figure 1). The delays were notably long (culling: 2.67 days; disposal: 3.45 days) prior to the epidemic peak in late March (figure 1*b*,*c*) despite the initially low number of IPs (figure 1*a*) and relatively low operational demand on either control effort (electronic supplementary material, figure S1b,c). The mean culling delay increased around late February and then steadily declined until mid-April (figure 1*c*). The disposal delay was highest at the start of the outbreak, and decreased dramatically in late April (figure 1*c*), coinciding with the epidemic being brought under control (figure 1*a*). When the 24 h culling policy was implemented after 1 April, these declined to 1.46 and 1.8 days, respectively. The longest recorded delays (culling: 40.6 days on 10 March; disposal: 41.5 days on 17 March) occurred at the start of the epidemic (figure 1*b*). The number of farms that experienced delays exceeding one week decreased as well after 1 April (for culling: 31–4; for disposal: 58–55). There was no correlation between the culling and disposal delays on individual farms (*ρ* = 0.07). Disposal delays approximate a long-tail distribution (inset in figure 1*c*), such that a wait-time exceeding 3 days (34.9% of disposal activities compared with 9.4% of culls) was as probable as less than 1 day (34.6% of disposal activities compared with 23.9% of culls). This partially reflects two distinct management phases: after 24 April, disposal activities, which previously consumed more operation time (3.21 ± 0.08 days delay) than culling activities (2.23 ± 0.06 days delay), were observed to be markedly more efficient (0.53 ± 0.03 day delay compared with 1.35 ± 0.05 day delay) (figure 1*c*). In other words, before late April, infected live animals were kept on IPs for a long time and their carcasses for longer; after this period, depopulation of IPs tended to happen more quickly and carcass disposal quicker still.

Cox proportional hazards regression models revealed that the size of infected farms was positively correlated (*p* < 0.01) with both the culling and disposal delays at the individual farm level (table 1). We note that the size of infected farms was consistent nationally throughout the entire epidemic (electronic supplementary material, figure S1a). Overall, with control demand and farm density held constant at mean values, an addition of 100 livestock reduces the daily rates of their respective activities by factors of 0.966 (that is, a decline of 3.7%) and 0.978 (2.2%) on average. Estimation of survival functions for farms grouped by size also showed as much as a 109% increase in the chances that local intervention remained incomplete after 48 h if the premises contains 500 livestock or more (electronic supplementary material, figure S2). A comparison of HRs summarized in table 1 found that the effects of farm size on both control measures were in qualitative agreement between the two subdivided time frames before and starting on 1 April. When the effects of control demand were evaluated over the full epidemic timeline, they correlated with drops in both culling and disposal efficiencies. In common with farm size, control demand constrained culling activities more heavily than it did for disposal activities. For every 10 pending actions, the daily culling rate was reduced by a factor of 0.87 (a decrease of 13%) and the daily disposal rate by 0.916 (8.4%) (table 1). However, for farms reported prior to 1 April, increased levels of control demand did not produce a statistically significant change to the durations of their disposal delay. Furthermore, culling delay was notably shortened with rising control demand, contrary to our expectation that wait-time would be lengthened when operational capacity is under increased strain. While counterintuitive, this result suggests that culling operations prior to policy reinforcement may have been delayed predominantly by factors related to management ‘awareness’, i.e. initially slow reaction to contain the outbreak when reports were scarce, instead of by caseload competition. The number of neighbouring IPs, i.e. farm density, was also consistent over time (electronic supplementary material, figure S1d), but unlike farm size, its operational impacts were in general found to be statistically insignificant (table 1). Only prior to 1 April did the covariate show a moderately significant effect, when an addition of 10 infected neighbours within a 5 km radius increased daily culling rate by a factor of 1.087 (8.7%).

**Table 1.** Survival analysis of response delays on infected premises (IPs). 1 April 2001 represents the date on which national control policy was strengthened by the inclusion of a 24 h target window for culling IPs following case report.

case report date	factor	culling delay		disposal delay	
		hazard ratio (95% CI)	*p-*value	hazard ratio (95% CI)	*p-*value
before 1 April	farm size	0.973 (0.963–0.984)	0.00	0.984 (0.973–0.994)	0.002
	control demand	1.183 (1.132–1.235)	0.00	0.988 (0.973–1.004)	0.14
	farm density	1.087 (1.020–1.159)	0.011	0.968 (0.903–1.039)	0.37
on or after 1 April	farm size	0.957 (0.947–0.968)	0.00	0.977 (0.968–0.987)	0.00
	control demand	0.883 (0.848–0.921)	0.00	0.889 (0.876–0.902)	0.00
	farm density	1.031 (0.974–1.093)	0.29	1.012 (0.949–1.078)	0.73
entire timeline	farm size	0.966 (0.959–0.973)	0.00	0.978 (0.971–0.985)	0.00
	control demand	0.870 (0.852–0.889)	0.00	0.916 (0.907–0.926)	0.00
	farm density	1.041 (0.997–1.086)	0.07	0.952 (0.909–0.998)	0.041

Model comparison using the likelihood-ratio tests further supported farm size and control demand as significant contributing factors of response delays (electronic supplementary material, table S1). In our ‘policy agnostic’ analysis under the time-independent response, we predicted delays conditional on both covariates; the same applies when we assume different hazard rates before and after 1 April under the time-dependent response, with the exception of the disposal of farms reported before 1 April, which is modelled to be delayed independent of control demand. Parametric bootstrap and graphical inspections showed that our delay predictions captured the general properties (effect size and direction) of the observed data (table 2; electronic supplementary material, figures S3 and S4).

**Table 2.** Comparisons between the recorded and the predicted delays on infected premises (IPs). *Left data column*: survival analysis using mostly two-factors Cox proportional hazards regression models for IPs reported across the specified epidemic timeline. *Right data column*: parametric bootstrap analysis using predictions generated with resampled covariate values.

	factor	case report date	from the recorded delays		from the predicted delays
			hazard ratio (95% CI)	*p-*value	mean hazard ratio (s.d.)
culling	farm size	entire timeline	0.965 (0.958–0.973)	0.00	0.965 (0.001)
		before 1 April	0.972 (0.962–0.983)	0.00	0.972 (0.002)
		on or after 1 April	0.957 (0.946–0.967)	0.00	0.957 (0.002)
	control demand	entire timeline	0.873 (0.855–0.891)	0.00	0.872 (0.013)
		before 1 April	1.180 (1.130–1.231)	0.00	1.180 (0.017)
		on or after 1 April	0.885 (0.849–0.923)	0.00	0.885 (0.017)
disposal	farm size	entire timeline	0.979 (0.972–0.986)	0.00	0.979 (0.001)
		before 1 April	0.984 (0.974–0.995)	0.003	0.984 (0.002)
		on or after 1 April	0.977 (0.968–0.987)	0.00	0.977 (0.001)
	control demand	entire timeline	0.916 (0.906–0.925)	0.00	0.915 (0.006)
		before 1 April	0.988 (0.973–1.004)	0.15	**—**
		on or after 1 April	0.889 (0.877–0.902)	0.00	0.889 (0.006)

**Figure 2.**
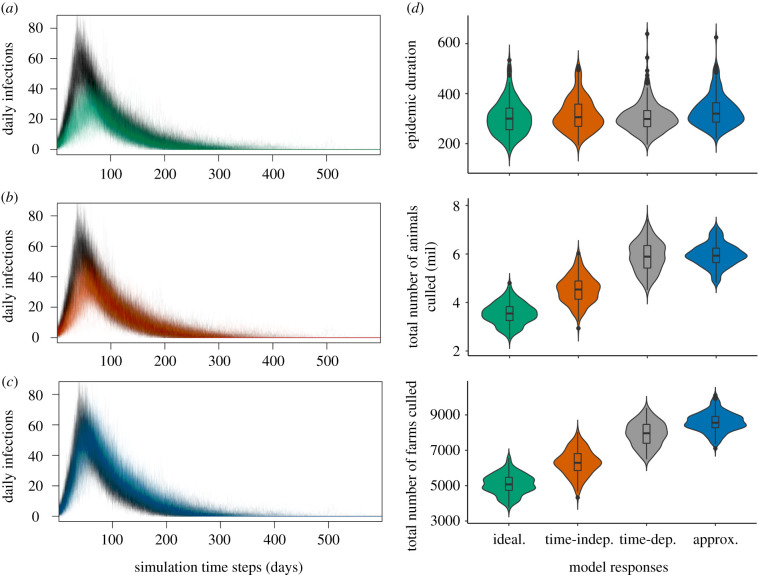
Simulations of daily FMD incidence time-series and overall management success in the extended Warwick model with variable culling and disposal delays on individual farms. The reference dynamics, shown in black (*a*–*c*), represent epidemic profiles conditional on locally heterogeneous delays as a function of farm size, control demand and policy timeframes (time-dependent response). The resulting dynamics of fixed, idealized response (*a*) and randomly drawn, approximated responses (*c*) are shown in green and blue, respectively. The time-series in orange (*b*) illustrate the changes in the dynamical pattern after removing the 1 April policy reinforcement factor from model description (time-independent response). Two hundred simulations were run per model, each initialized at 1 February 2001 and continued until disease elimination. The management outcomes of the model responses are shown in corresponding colours using violin plots (*d*) under three standard measures of control effectiveness: epidemic duration, total number of animals culled and total number of farms culled.

When variable culling and disposal delays on individual farms based on logistical factors and control policy are included in the Warwick FMD simulation model, we observed changes in the epidemic profile as well as significant differences in the overall epidemic size, quantified here by the total numbers of animals and farms (i.e. combination of IPs and DCs) culled (figure 2*a–d*). On the other hand, the durations of the epidemics were not markedly affected, which averaged between 305 and 332 days across all four management scenarios (figure 2*d*).

Simulations that include response times as a function of farm size, control demand and policy timeframes, i.e. time-dependent response, generally led to (i) steeper initial epidemic growth, (ii) earlier epidemic peaks and (iii) larger peak sizes. We also observed more frequent recurrences of small outbreaks toward the end of the epidemic (figure 2*a*–*c*). Assuming an idealized response process, exactly consistent with the policy recommendation from the start, results in the lowest number of animals and farms targeted for depopulation (approx. 59% of the maximum expected values), the slowest growth rate and typically fewest daily infections throughout the epidemic (figure 2*a*,*d*). Ignoring the different delay patterns before and after the reinforcement of control guideline on 1 April, i.e. time-independent response, generated slower increases in daily incidence and reductions in epidemic size (figure 2*b*,*d*). The trajectories of incidence decline are largely consistent with those under the time-dependent response. By contrast, drawing random delay times from the empirical delays irrespective of policy and logistical constraints results in incidence time-series that more closely follow the operationally informed predictions from the time-dependent response, particularly during the growth phase of the epidemic, but is also characterized by slower deceleration after the peak (figure 2*c*). Overall, under the random delay time scenario, the distributions of animals and farms culled have slightly higher means and smaller variances compared to the results under the time-dependent response (figure 2*d*).

## 4. Discussion

Our statistical analysis of times between infection reports, culling completion and disposal completion during the 2001 FMD outbreak in the UK showed significant farm-level variation in response delays. Response delays were initially long, but shortened over time with the intensification of control measures and the decline in disease incidence. Nevertheless, culling delays on IPs generally failed to meet the specified 24 h target. Efforts to depopulate several IPs were delayed for more than two weeks. Under strict movement restriction, this would not only raise concerns over animal welfare, but may substantially add to the overall economic cost due to the increased risks of mass welfare culls [45]. In addition, long culling delays may promote recurrent outbreaks in formerly controlled regions [46] and long-distance airborne spread if they occur during particular atmospheric conditions [47]. Compared to culling, the response times for carcass disposal were considerably longer during the early phase of the epidemic before they abruptly shortened in late April. In a general outbreak of livestock disease, a similar pattern of slow control action downstream to culling (e.g. carcass removal) during the peak epidemic period could potentially undermine the management objectives.

Survival analysis of response times shows a dependency of culling and disposal delays on temporal and demographic variables. In particular, farm size, measured by livestock quantity, correlated positively with both types of delays, suggesting a tradeoff between the scale and the efficiency of control at the individual farm level. Slower response to large farms raises a potentially important concern for global agribusiness and livestock production: while large farming units may yield higher productivity, they could also create a logistical bottleneck for control actions in the event of an outbreak. This adverse effect further suggests that large farms not only have a dominating role in the risk of onward transmission [48,49], but may also increase the potential for pathogen exposure on susceptible premises due to infective hosts (i.e. live animals or carcasses) being kept longer onsite. Future management strategies may thus be able to slow epidemic growth by prioritizing the treatments of farms with high livestock counts.

Increased control demand, represented by the accumulation of untreated cases and farms not yet disposed, was found to create significant operational delays for the majority of the epidemic. Thus, a sudden surge of infection reports that accelerates epidemic spread may concurrently hinder local containment effort, complicating the management challenges. This dynamical interaction further suggests a positive feedback between management and disease spread: lagged responses to infected farms increase transmission opportunities, which results in greater number of infections and more belated responses. Therefore, response delays at earlier stages of an epidemic can be compounded into longer delays at later stages along the epidemic curve, which is consistent with previously observed patterns of large-scale FMD intervention [22]. This management implication reinforces the importance of allocating sufficient resources toward pre-emptive responses such as disease prevention (e.g. national prophylactic vaccination campaigns) and surveillance, and expeditious control actions early on in the outbreak. Given that control demands and delay times are fundamentally uncertain before a response is initiated, planning of response might consider strategies that are robust to these uncertainties initially [33], but can adapt once the operational load is known and the associated delays are realized [50].

Contrary to expectation, infected farm density (i.e. number of neighbouring IPs) did not appear to strongly affect the speed of either stage of control action. This result, in combination with the effect of farm size, may inform the development of more protective farming practices against epidemics of livestock diseases. For instance, as many countries move further towards industrialization and urbanization, land policies that favour a dense distribution of small farms over a sparse distribution of large operations may enable more expedited intervention in the event of an outbreak. However, spatial clustering promotes epidemic spread [51]; hence, planning for outbreak response conditional on the landscape-level distribution of farms of different sizes will allow a more tailored response to outbreaks when they occur. The generality of these logistical effects and their practical applications is an area we intend to study in the future.

Few models of epidemic management in livestock populations have attempted to explicitly account for variable response delays as a function of logistical factors. Early predictions of outbreak and management success were commonly made on the assumptions of spatially uniform or time-invariant delay. In recent years, individual-based simulations introduced logistical constraint by limiting the number of daily control actions [30,52,53]. While this description accounts for control demand and allows different delays when there is an excess number of target premises, the control capacity is often determined arbitrarily and unaffected by the number of animals on individual farms. Large-scale, stochastic livestock models such as AusSpread [54], AADIS (Australian Animal Disease Spread) [55], NAADSM (North American Animal Disease Spread Model) [56] and InterSpread Plus [57] are capable of modelling spatially heterogeneous response delay, but the embedded delay functions have typically been underpinned by expert opinions instead of empirical data. Compartmental models fitted to historic outbreak data [20,58] have predicted alternative time-series of known epidemics when they assumed different distributions of response times. However, the durations of delay were not explicitly linked to farm attributes or disease dynamics. A recent model of human disease by Tao *et al*. [59] combines a spatial compartmental model with an individual-based simulation to describe a more realistic response process regulated by spatio-temporally varying logistical constraints—drained resources and high density of control targets confer extensive delay in the local response. The model's ability to inform the management of livestock disease outbreaks is nevertheless limited; its assumption of identical control units omits demographic heterogeneity that may be used to describe important farm-level variations in enclosure capacity, holding practices and species compositions, among other covariates.

Our simulations explicitly account for the timeliness of individual control actions and integrate disease dynamics with management dynamics at both the local and national levels. By simulating epidemic scenarios under different descriptions of response delays using the Warwick model, we demonstrated that misrepresenting response efficiencies may lead to biased outbreak projections. The assumption of idealized delays predicted the lowest epidemic impacts on a national scale. Thus, an optimistic assumption of universally prompt responses would drastically underestimate the magnitude of the resulting outbreak. Accounting for realistic logistical constraints and a policy timeline based on observed lags resulted in more severe outbreak projections. The range of predictable outcomes further depends on the model's capacity to recognize, isolate and quantify patterns of early-stage intervention that may comprise slow, under-coordinated responses to initial detections amidst a novel outbreak. Interestingly, when we sampled case-specific delays randomly from their full empirical distributions without any operational knowledge, we were able to approximate the outbreak dynamics generated by the most informed model, suggesting that an assumption of variable delays alone may enhance model prediction even in the absence of spatial or temporal details. This alternative model, a potentially convenient approach for retroactive data analyses, is nevertheless impractical to adopt in real-time forecasts; when the complete pattern of operational delays has not yet emerged, it may support an oversampling of early response times and conceal the operational reality that becomes apparent only during later stages of intervention. In general, the overall epidemic severity increases with the level of variation we incorporate into model responses.

The direction and magnitude of forecast bias may lead to sub-optimal management recommendations when comparing culling-based interventions to, for example, frequently debated vaccination-based interventions [33,60]. The degree to which these biases will result in incorrect management recommendations is beyond the scope of this analysis; however, this work highlights the importance of accounting for operational, as well as epidemiological, uncertainties and their potential impact on management recommendations as well as epidemic forecasts [61]. The measures our simulation used for predicting management outcomes (i.e. total animals and farms infected, epidemic duration) commonly represent conflicting, difficult-to-resolve objectives between policy makers and multiple stakeholders [48,53]. Therefore, resolving uncertainties in local response delay can also potentially reduce the need for time-consuming debates on management strategies by meeting multiple objectives at once.

While top-down policy reinforcement is critical to overall management success, our results emphasize the additional need for policy makers to base their expectations of response efficiency on realistic logistical constraints. The impacts of logistical constraints may be magnified in countries deficient in economic, diagnostic and operational capacity. Should the infectious agents remain highly transmissible in decomposing carcasses and be capable of airborne propagation, then the risk of extensive delays during mass disposal may raise even greater concerns [62]. Therefore, quantitative assessment of control logistics during novel outbreaks can be invaluable in the development of appropriate intervention strategies, including optimal resource allocation and pre-emptive planning. Providing rapid operations research in tandem with epidemiological observations allows management to better anticipate needs ahead.

Our current study can be extended such that each new case report is weighted according to the number of days it remains untreated in that period, giving a possibly more realistic description of control demand adjusted for urgency. We may also evaluate response delay relative to different temporal boundaries: incorporating the start dates of a control action enables measurements of (i) the delay to initiate desired action and (ii) the amount of time spent on said action, from which we can calculate finer-scale patterns such as variation in local handling time per animal. Given that the aim of our study is not to recreate the 2001 outbreak, our analysis uses IP as proxy for control demand without modelling the scheduling effects of DC/CP and other culls. However, similar pre-emptive control measures, including concurrent vaccinations (as was used in the 2001 FMD outbreak in The Netherlands), might be considered in future studies that intend to characterize the entirety of an operational backlog (see [32]). We note that response wait-times may also be strongly shaped by farm-level covariates not explored in our survival analysis, including elevation, species composition (e.g. the ratios of sheep to cow) and accessibility measured by the shortest distance to the nearest road. Our study assumes no supplementation of resource with increasing caseload, yet conceivably the latter might trigger ‘stronger’ responses in the forms of new financial commitment made to control programmes, resource reallocation and other changes in operational capacity over time. Therefore, global covariates such as the amount of available resources (e.g. personnel, funding, public support) and the number of epidemic foci may also be useful to consider. Investigating the logistical impacts of these covariates will broaden our understanding of context-dependent delay and help tailor management strategies to particular disease systems and geographical regions.

## Data accessibility

All simulation outputs are stored in the Dryad Digital Repository (http://dx.doi.org/10.25349/D9P315). R code for statistical inferences, model predictions and figure generations (excluding confidential farm demography data) are available at the following repository: https://github.com/taoyun/fmd_responses. Data on the 2001 UK FMD outbreak and response processes are accessible upon request from the Department for Environment, Food and Rural Affairs (DEFRA) of the government of the United Kingdom. DEFRA contact information is listed at the following website: https://www.gov.uk/government/organisations/department-for-environment-food-rural-affairs.
